# Integration of ESG Information Into Individual Investors’ Corporate Investment Decisions: Utilizing the UTAUT Framework

**DOI:** 10.3389/fpsyg.2022.899480

**Published:** 2022-04-25

**Authors:** So Ra Park, Kum-Sik Oh

**Affiliations:** ^1^Department of Tourism Management, Cheju Halla University, Jeju, South Korea; ^2^Division of Global and Interdisciplinary Studies, Pukyong National University, Busan, South Korea

**Keywords:** UTAUT, ESG criteria, investment decisions, individual investors, ESG information integration

## Abstract

Environmental, Social, and Governance (ESG) criteria are now considered significant, global non-financial evaluating factors of corporate value. However, no attention is given to what influences the integration of ESG information by individual investors in their investment decisions. This study first identifies different types of information investors use to make investment decisions. Risks identified in information integration in investment decision making is reviewed. Next, the Unified Theory of Acceptance and Use of Technology (UTAUT) model is used to identify individual investors’ investment tendencies and the factors affecting integration of ESG information into investment decisions. Each of four categories for UTAUT innovation adoption factors (performance expectancy, effort expectancy, social influences, and facilitating conditions) are discussed in relation to how they affect individual investors’ integration of ESG information. Standardization of ESG reporting and evaluation frameworks would reduce efforts to adopt ESG information and could build a strong foundation for facilitating ESG information integration. Corporates’ efforts to further communicate their ESG management through their investor relations and active governmental well as non-governmental organizations’ participation are recommended.

## Introduction

Investors use various strategies to gain quality information when making investment decisions. Investors traditionally make decisions based solely on financial performance, but they now have more goals than simple financial gain. Also, they are using more than just financial information (such as ESG information) to make investment decisions (Sultana et al., 2018). With regard to the investment using ESG information, Sustainable and responsible investment (SRI) is an investment approach that “integrates ESG factors in the research, analysis, and selection process of securities within an investment portfolio in order to better capture long-term financial returns for investors, and to benefit society by influencing the behavior of companies” (Eurosif, 2021). Investors can influence corporate CSR behaviors and management (Park and Ghauri, 2015). Therefore, positive social and environmental outcomes as well as long-term financial gains necessitate integration of ESG information by investors.

To attract individual investors, companies need to expose themselves to individual investors more since they tend to invest in familiar companies (Barber and Odean, 2013), and companies need to present their data a format easily accessible and digestible. While three are issues regarding standardization and comparability exists, policy makers and regulators try to improve corporate ESG disclosures and the standardized rating agencies’ reports. For example, the Investor Advisory Committee (IAC) under the SEC operates to ensure availability and usability of data for individual investors. Also, there is an increasing number of nations mandate that companies disclose their ESG management practices.

Despite the growing desire of individual investors’ for ESG investment, research on ESG information integration by individual investors is insufficient. In addition, even for active involvement in SRI, it is necessary to investigate the factors that influence individual investors to integrate ESG information. Therefore, the objective of this paper is to explore individual investors’ intentions to integrate ESG management information and the ways they actually integrate it into their investment decisions. By doing so, this study contributes to the literature in two ways. First of all, previous studies on ESG information integration were mainly focused on institutional investors, but this study focuses on individual investors. Thus, it contributes to expanding the understanding of factors that encourage ESG information integration into the investment decisions of individual investors and closes the gap in research that intensively studied institutional investors. Second, this study meets the current demand for understanding ESG information integration. This study analyzes ESG information integration by individual investors by utilizing a traditional method, a risk management perspective, and the UTAUT innovation adoption model. In previous studies, the UTAUT model was mainly used for technical knowledge or information, but it is designed for use with any type of innovation in various disciplines. Thus, this study contributes to expanding theoretical discussion of the UTAUT model.

The organization of this paper is as follows. “Information that Individual Investors Use” discusses the variety of information that individual investors use when making investment decisions. “Discussion of ESG Information Integration Using Risk Management View” brings the risk management view into investment decision making to identify the risks posed by ESG information integration. In “Discussion of the UTAUT for Integration of ESG Information in Investment Decisions”, factors influencing individual investors’ integration intentions are identified by using the UTAUT model. Lastly, the paper presents the conclusions from, and the contributions, implications, and limitations of, this research.

## Information That Individual Investors Use

Information enables investors to manage risks associated with investment decisions (Heukelom, 2007). Investment decision making is a continuous effort to reduce the level of uncertainty/risk, and acquisition of good information and proper analysis of information can help the process (Danarti et al., 2020). Individual investors’ presence in the stock market is increasing. Individual investors account for roughly 25% of stock market activities due to the market volatility created by COVID 19, which is up from 10% of stock market activities in 2009 (Winck, 2022). Due to the increasing importance of individual investors in financial markets, we investigate and discuss the variety of information that individual investors integrate into their investment decisions. Table 1 shows the types of information integrated by investors for their investment decisions, as presented in previous studies.

**Table 1:** Information integrated by investors into investment decisions.

Study	The source of information	Objective	Key findings
Obamuyi, 2013	Past performance of the company’s stock, expected stock splits/capital increases/bonuses, dividend policies, expected corporate earnings, and get-rich-quick schemes	Determine the main factors influencing investment decisions of investors	Past performance of the company’s stock, expected stock splits/capital increases/bonuses, dividend policies, expected corporate earnings, and get-rich-quick schemes are the most influencing factors on investment decisions in Nigeria. The investment climate and the market environment can be made friendly and conducive to attracting investors by creatively developing programs and policies that impact investors’ decisions in order to maximize the value of firms and to enhance the wealth of investors
Pradhan and Kasilingam, 2015	Five corporate actions such as dividend announcement, bonus announcement, right issue, buy back and stock split issue	Find out the most influential corporate actions on investment decisions of individual investors	Dividends have the highest influence on investment decisions, and stock splits have the lowest influence. Demographic factors significantly influence the announcement-based investment decision. This encourages investors to hold shares for a long period and is more relevant to the market than other announcements
Hafenstein and Bassen, 2016	Sustainable information (ESG)	Identify the factors affecting the use of ESG information and investment decisions in corporations	Investment decisions are influenced by individual sustainability orientation, and non-professional investors could not distinguish between various aspects of sustainability (that is, ESG criteria). Thus, companies need to inform investors about sustainable practices
Amel-Zadeh and Serafeim, 2018	ESG information	Investigate the reasons for the use of ESG information by investors	The main reason investors consider ESG information when making investment decisions is because they think it is important for investment performance. ESG information is considered to mainly provide information on risks, but it is difficult to use ESG information due to a lack of reporting standards
Uslu Divanoglu and Bagci, 2018	Situations of individual & social, the level of basic knowledge and general factors	Identify the factors influenced by individual investors, and the factors influencing decisions when making investment decisions	Situations of individuals and society, the levels of basic knowledge, and other general factors influence bank investment decisions
Sultana et al., 2018	ESG issues	Investigate the impact on investment decision making of individual stock market investors’ preferences for ESG issues and their investment purposes	ESG issues affect investment decisions and are the purpose of investment
In et al., 2019	ESG data	Investigate how to evaluate the quality of ESG data to facilitate its usage by investors and its integration in investment decision making	The quality of ESG data is ultimately determined by the investment decisions in which such data are used
Jang and Park, 2019	Domestic and global factors, such as the global recession and geographical risks	Investigate the determinants of global investors’ investments in Korean treasury bonds	Investors with short-term investments are more sensitive to domestic and global factors. Investors with long-term investments are more sensitive to international factors, such as a global recessions and geo-political risks
Khemir et al., 2019	ESG information	Investigate whether investors use ESG information to choose investments in the Tunisian capital market	ESG information influences investment decisions in Tunisia, and governance and social information have more influence than environmental information. Thus, corporations should pay more attention to ESG information disclosure practices
Lee et al., 2020	ESG information	Investigate the differences in performance and risk between high and low ESG investment portfolios	ESG integration helps avoid risks arising from ESG investment. Portfolios with high ESG ratings continuously lower the risks along with providing excellent performance, compared to portfolios with low ESG ratings
Ullah et al., 2021	Corporate CSR strategy	Investigate the effect on investors’ investment decisions from the concept of corporate social responsibility (CSR)	Corporate CSR strategy plays a critical role in forming investor investment behavior
Prajapati et al., 2021	ESG and credit ratings	Find out the main drivers and factors that influence individual investors’ investment decisions in green bonds	ESG ratings and green bond issuers’ credit ratings are the main factors influencing individual investment decisions

One of the critical pieces of information affecting individual investment decisions is publicly available information affecting stock prices; thus, information regarding product safety and quality, corporate ethics, employee relations, community engagement, and organizational environmental activities are in high demand by investors (Chandra and Kumar, 2012). A corporate announcement is useful public information helping individual investors to make the right investment decisions efficiently and at the right time (Pradhan and Kasilingam, 2015).

Financial information driven by various analyses is utilized to determine investment risks and to find investment opportunities (Nur Ozkan-Gunay and Ozkan, 2007). Investors use financial ratios to avoid default risks and maximize financial leverage, to balance long- and short-term investments, to ensure debt coverage stability, etc. To enhance the predictive power of forecasting, it is necessary to explore other non-financial factors (Lin et al., 2011; Harford and Uysal, 2014). Also, non-financial macroeconomic indicators have been found to correlate with investment returns when data from a number of countries are analyzed (Ang and Piazzesi, 2003; Mahmood and Mohd Dinniah, 2007; Pramod Kumar and Puja, 2012). Many investment decision studies have dealt with non-financial information, such as political environments (Herbst and Slinkman, 1984), geopolitical risks (Kim, 2011), consistency in economic policies (Jang and Park, 2019), and legal issues (Dincer, 2007; Jomini, 2011). ESG criteria are the most actively researched non-financial factors to consider (along with financial information) in assessing the investment attractiveness of a company (Velte, 2019; Lee et al., 2020). Information on the ESG criteria themselves includes corporate ESG management disclosure, rating agencies’ ESG ratings, news regarding corporate ESG activities, and more.

ESG investing is stimulating mainstream interest from individual investors for two reasons. First, ESG investing actively promotes ethical investment practices; second, ESG investments are considered a means to improving the performance of managed portfolios, and a way to increase returns and reduce portfolio risk (Broadstock et al., 2021).

## Discussion of ESG Information Integration Using Risk Management View

Due to the lack of research regarding what causes resistance in individual investors to ESG information integration, a review of recent literature on information integration in investment decision making was conducted (Table 2). The review provides the issues in integration of information. According to Chavas (2004), there are three main sources of risk: (1) when the causes of events are difficult to control or measure precisely; (2) when decision makers lack the ability to process information regarding investment outcomes from the given options; and (3) when the necessary information is too costly to obtain or process. Since the first source cannot be easily controlled or identified, risks are being managed by lowering information processing costs and increasing the quality information acquisition. Each of the studies in Table 2 is categorized in terms of types of information risks.

**Table 2:** Information integration issues using the risk management perspective.

Study	Theoretical lense	Information management focus	Type of investors related	Key finding
Jain et al., 2015	Prospect theory, behavioral theory	Information gathering/processing difficulty	Individual investors	Uncertainty creates bias in individual investors due to the unpredictability
Dong et al., 2016	Price informativeness	Information processing cost	All investors	Information processing costs reduce firm-specific acquisition costs
Pennington and Kelton, 2016	Information processing theory	Information processing cost	All investors	Individual investors use different stopping rules when collecting the information necessary for decision making
Danarti et al., 2020	Prospect theory, risk-taking bias, loss-aversion	Information gathering/processing difficulty	Individual investors	Uncertainty brings in heuristics to minimize the effort in making investment decisions, introducing bias
Blankespoor, 2019	Mosaic theory, disclosure theory	Information processing cost/information standardization	All investors	Using Extensible Business Reporting Language brings more disclosures
Blankespoor et al., 2019	Information awareness and acquisition costs	Information processing cost	Individual investors	Information awareness and acquisition costs deter individual investors from using accounting information
Alaaraj and Bakri, 2020	Financial literacy	Information gathering/processing difficulty	All, but applies more for individual investors	Financial literacy (knowledge and awareness) influences investment decision making
Griffin et al., 2020	Impossibility of informationally efficient market	Information processing cost	All investors	Including disclosures of environment information increases information processing costs for analysts, making it harder for investors to include many firms with ESG management in the portfolios
Huang et al., 2021	Impossibility of informationally efficient market	Information processing cost/information standardization	All investors	Extensible Business Reporting Language reduces information processing costs
Yi et al., 2021	Information asymmetry	Information processing cost/information standardization	All investors	Comparability of financial statements reduces information asymmetry and makes companies issuing an IPO more attractive for acquisitions and joint ventures
Chen et al., 2022	Information processing cost	Information processing cost	All investors	Low information-acquisition costs for financial analysts increase information production, improve forecast accuracy, and result in better recommendations
Kim and Gamble, 2022	Risk and uncertainty	Information gathering/processing difficulty	All investors	Under uncertainty, information acquisition becomes harder for investors with low analytical abilities, resulting in higher reward estimations

In accordance with the Chavas’s risk management view, integration of ESG information by individual investors is also related to quality information and information processing costs. Lack of comparability in the reported corporate ESG activities and corporate greenwashing of ESG performance make it harder to collect, analyze, and compare ESG information (Amel-Zadeh and Serafeim, 2018). The divergence in ESG ratings introduces uncertainty in decision making (Berg et al., 2019). Lack of standardization in ESG disclosures and in reporting frameworks and measurements was mentioned by Amel-Zadeh and Serafeim (2018) as the main problem for ESG information integration. Individual investors’ lack of skills in acquiring and processing ESG information adds to the information processing costs. While there are differences, individual investors lack financial literacy (Alaaraj and Bakri, 2020) and incur high costs from information awareness and acquisition (Blankespoor et al., 2019).

ESG information integration is a new trend for investment decisions nowadays. Innovation is defined as the development (generation) and/or adoption of new ideas or behaviors (Damanpour and Schneider, 2009). Therefore, integration of ESG information in order to make investment decisions can be considered an innovation. Successful ESG integration in investment decisions needs a new perspective outside traditional risk management. Therefore, the following section presents an innovation adoption perspective to explore the various factors affecting investors’ integration of ESG information. There are multiple innovation adoption and integration models (Compeau and Higgins, 1995), but the UTAUT model is the most comprehensive and tested model (Venkatesh et al., 2003). The UTAUT model has been used in previous research related to investment integration/adoption (Gunawan and Novendra, 2017; Francisco and Swanson, 2018; Sun et al., 2019; Gupta et al., 2020; Christensen et al., 2021).

## Discussion of the UTAUT for Integration of ESG Information in Investment Decisions

**Figure 1:**
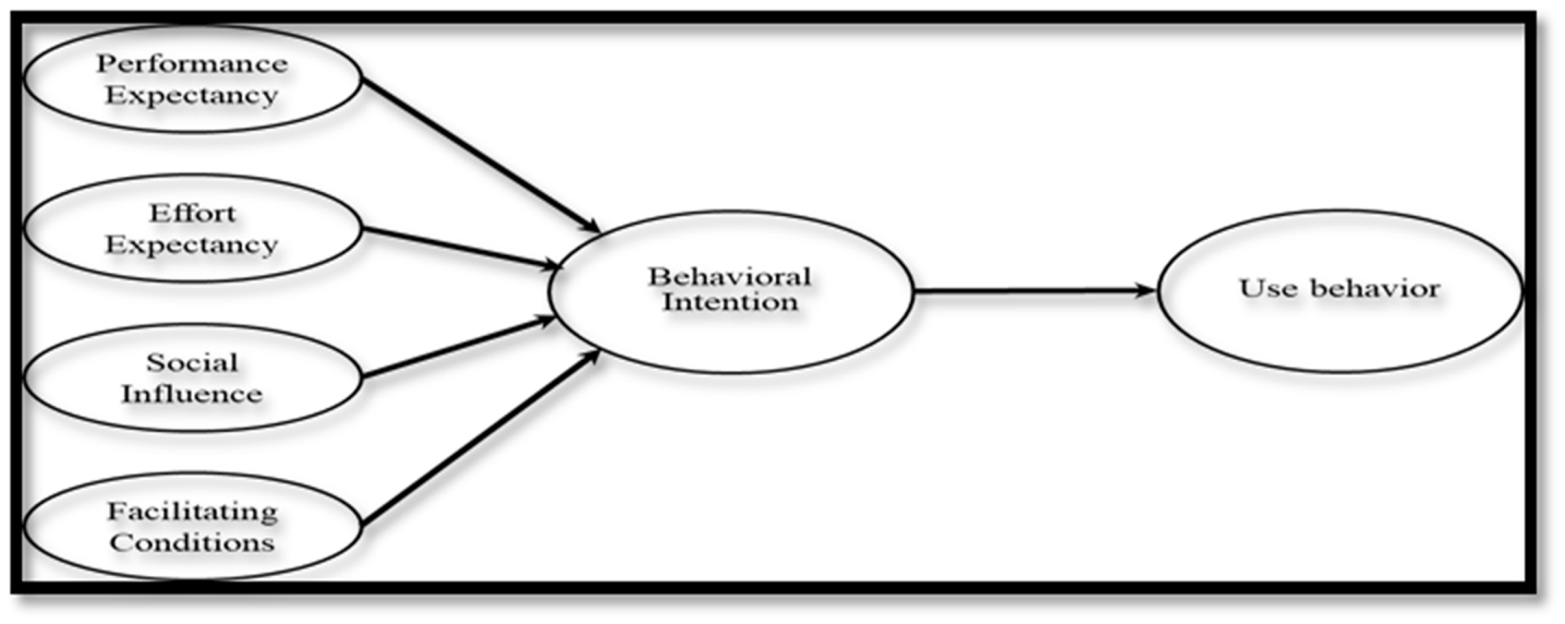
The UTAUT model (Venkatesh et al., 2003).

UTAUT was developed incorporating eight different acceptance models under four main theoretical backgrounds, and it is used to understand the intentions for and use of innovation. The comprehensive model has been tested vigorously and strongly backed by multiple theories, and thus, this study adopts it to explore various aspects of investors’ integration of non-financial information into their decision making (Venkatesh et al., 2003). The UTAUT proposed four categories: performance expectancy, effort expectancy, social influence, and facilitating conditions (Venkatesh et al., 2003, see Figure 1). The current paper adopts these categories to explain ESG information integration, which is defined as individual investors “explicit inclusion … of ESG risks and opportunities into traditional financial analysis and investment decisions based on a systematic process and appropriate research sources” (Eurosif, 2014).

One of the main goals of SRI through ESG information integration is long-term returns for investors (Eurosif, 2021). Performance expectancy, the first component of the UTAUT model to explain ESG information integration by individual investors, is based on the potential users’ belief that the adoption of innovation or innovative behavior is expected to bring better performance. Information on how firms manage their ESG issues is valuable in predicting the firms’ long-term sustainability. Investors adopt different kinds of strategies using ESG information, and they always seek good performance indicators for future corporate sustainability. ESG investment is largely motivated by the promise of positive performance (Plagge and Grim, 2020). Therefore, information regarding positive corporate ESG performance should become important corporate ESG information that will positively influence individual investors’ integration of it into their investment decisions. A number of studies in the literature have suggested that companies with good ESG practices have a higher return on investment. Friede et al. (2015) conducted a study on ESG/SRI factors and found a significant positive relationship between ESG performance and financial performance. Abate et al. (2021) shows that fund portfolio composed with high ESG-rating securities performed better that low ESG-compliant counterparts.

The second component comprising the UTAUT model is effort expectancy, the ease of integrating ESG information in making investment decisions (Venkatesh et al., 2003). The expectancy is related to how much potential users of ESG information think the entire process will be easy, flexible, and understandable. Eccles et al. (2014) discussed investors’ lack of required knowledge or training to use ESG information to do the job. Disclosure processing costs (Blankespoor et al., 2020) should negatively influence effort expectancy. According to Kempeneer et al. (2021), rating agents provide too divergent ratings to rely on. Therefore, individual investors are likely to have a negative effort expectancy regarding ESG integration due to their limited resources in processing disclosures as well as the divergence in corporate ESG disclosures and ESG ratings. Negative effort expectancy will deter individual investors from integrating ESG information into their investment decisions.

Thirdly, social influence in the context of ESG information integration is defined as the level of perception potential investors have regarding how others believe they should use ESG information (Venkatesh et al., 2003). The biggest social influence seems to be the current business environment, which urges businesses to adopt socially responsible ways, and to promote all the stakeholders’ values. The places they live, their work, their cultures, and the surroundings in their living environments give individual investors information advantages increasing the chance of making positive performances (Ivkovic and Weisbenner, 2005; Massa and Simonov, 2006). Individual investors form investment-related knowledge and opinions from their physical environment and their online and offline communities. Because of their close communities, the investment community members (Haritha and Uchil, 2020) and individual investors often demonstrate herd behaviors (Olsen, 2008). They also rely heavily on information from financial online communities (Lerman, 2020). Research by Ammann and Schaub (2020) showed that postings from online communities affect individual investors’ decision making significantly—more so for investors who are smaller and less financially literate. Therefore, efforts on SRI involvement should be made in local investment communities where individual investors rely their investment-related information and form their investment ideas.

As the last factor influencing individual investor’s ESG information integration, facilitating conditions refers to the belief of individual investors have about the existence of legal, technical, and organizational infrastructures enabling integration and the actual existence of the conditions enabling facilitation. The most important facilitating condition for ESG information integration is legislation on ESG disclosure. Many companies in countries such as those in the EU and in South Korea need to report their ESG disclosures in the near future, and many other nations are expected to adopt such laws (Park and Jang, 2021). The domicile of the investor show integration of ESG information differently (Eurosif, 2014). The issue of ESG reporting and rating standardization is the most cited facilitating condition to be tackled (Eccles et al., 2017).

Discussing each of the four factors of ESG information integration reveals individual investors’ general as well as unique ESG investment tendencies. Promoting ESG information integration by individual investors gives a chance to better the quality of ESG disclosures, resulting in positive corporate performance (Raghunathan, 1999). Individual investors would integrate ESG information for their decision making only when they expect positive profitability and a low level of effort in processing the information. Individual investors’ participation in ESG investment will require their understanding of ESG management, which can be strengthened with investor relations. Utilizing corporate investor relations will enable individual investors to better integrate ESG information, and it will make them socially conscious investors in the long run. Most importantly, better facilitating conditions under proper laws and regulations, and standardization of the ESG frameworks and metrics are also required for ESG investment by individuals.

## Conclusion

This study identified the factors affecting individual investors’ integration of ESG information into their investment decisions. In this research, (1) we point out the importance of utilizing ESG information for investment decisions; (2) we identify the existing information integration problems for individual investors; (3) we extend the applicability of the existing UTAUT model in order to explain ESG information integration; and (4) we further promote strengthening corporate ESG management *via* individual investors’ adoption of ESG information.

While there is a great deal of academic attention given to understanding the adoption of ESG information by institutional investors (Eccles et al., 2017; Park and Jang, 2021), the existing research lacks an understanding of what encourages individual investors to integrate ESG information. Individual investors might not look resourceful as individuals, but their influence on financial market can be significant. Thus, this study contributes to our understanding of the factors that encourage ESG information to be integrated into investment decisions by individual investors, and it closes a gap in investor research that has been largely ignored. Additionally, this study contributes to expanding the generalizability of the UTAUT model by examining the ESG information integration by individual investors (a new research field).

The risk management perspective and the UTAUT model bring multiple factors enabling integration and potentially reducing integration barriers. Therefore, from a managerial perspective, companies can increase the quality of information and lower information processing costs for individual investors by providing quality ESG disclosures, inviting them to their IR meetings for in-depth Q&Rs, avoiding greenwashing, and following industry disclosure practices to increase comparability in their reports. Also, this study offers hints to finance-seeking companies on how to attract investment. First is to emphasize on positive corporate ESG performance to increase the expectations of individual investors. Second is to create comprehensible and comparable ESG reports for individual investors with limited resources. Third is utilizing social communities to attract potential investors. And finally, they can strive to standardize ESG reporting, evaluation frameworks, and ESG metrics. While there is no official standard provided for individual companies, there are standards used more frequently by industry. Merging these steps into industry practice will at least enable comparability within each industry. Also, the network externality each industry builds could influence what becomes standard. To sum up, ESG management is no longer a matter of choice, but an innovative process for investors’ investment decisions. Therefore, companies need to disclose not only their financial information but also non-financial corporate information such as ESG criteria. In other words, companies should learn to align their strategic purpose with social values and must efficiently allocate resources to meet the aim of sustaining ESG management.

This paper contributes significantly to current knowledge on ESG information integration by individual investors and provides practical implications for management. It can be expected that more such results can be obtained by applying different models and looking at ESG integration from a different perspective. The most apparent limitation of this research is the scope. It is aimed at encouraging individual investors’ integration of ESG information, and subsequent studies will need to examine the differences in ESG integration between institutional and individual investors.

## Data Availability Statement

The original contributions presented in the study are included in the article/supplementary material, further inquiries can be directed to the corresponding author.

## Author Contributions

SP conceived the idea for the manuscript. All authors contributed to the writing and development of the manuscript and have read and agreed to the published version of the manuscript.

## Funding

This work was supported by a Research Grant of Pukyong National University (2021).

## Conflict of Interest

The authors declare that the research was conducted in the absence of any commercial or financial relationships that could be construed as a potential conflict of interest.

## Publisher’s Note

All claims expressed in this article are solely those of the authors and do not necessarily represent those of their affiliated organizations, or those of the publisher, the editors and the reviewers. Any product that may be evaluated in this article, or claim that may be made by its manufacturer, is not guaranteed or endorsed by the publisher.
